# Empathy and Mentalizing of Mental Health Nurses: A Cross‐Sectional Correlational Study

**DOI:** 10.1111/inm.70002

**Published:** 2025-01-23

**Authors:** Gieke Free, Wilma Swildens, Adriaan Hoogendoorn, Aartjan Beekman, Berno van Meijel

**Affiliations:** ^1^ Altrecht Institution for Mental Health Care Utrecht Netherlands; ^2^ Inholland University of Applied Sciences Amsterdam Netherlands; ^3^ Department of Psychiatry Amsterdam University Medical Centre Amsterdam Netherlands; ^4^ Amsterdam Public Health Research Institute Amsterdam Netherlands; ^5^ Parnassia Psychiatric Institute Parnassia Academy The Hague Netherlands

**Keywords:** empathy, mental health services, mentalisation, psychiatric, psychiatric nursing, social work

## Abstract

In mental healthcare, therapists' empathy and mentalizing are associated with better opportunities to establish positive working relations with patients. The present study aimed to explore mental health nurses' level of empathy and mentalizing (compared with reference groups studying or working in different contexts), the association between mental health nurses' level of empathy and mentalizing and sociodemographic characteristics of these nurses, and the association between mental health nurses' level of empathy and mentalizing. A cross‐sectional design was used in adherence with the Strengthening the Reporting of Observational Studies in Epidemiology statement. The 28‐item Empathy Quotient was used to investigate empathy, and the 28‐item Mentalisation Scale was used to assess mentalizing. One hundred and seven mental health nurses working in different work‐intensity settings (intensive and intermittent‐intensive) participated in the study. Our analyses showed that mental health nurses had statistically significantly higher levels of empathy and mentalizing than the matched reference groups. They also showed that most mental health nurses' demographic characteristics were not statistically significantly associated with their level of empathy and mentalizing: not with age, years of work experience, or educational level. Only two aspects were statistically significantly associated: female gender (for higher levels of empathy and mentalizing) and practicing in an intermittent‐intensive work setting (for higher scores on the subscale ‘Motivation’ of the Mentalisation Scale). Furthermore, empathy and mentalizing of mental health nurses were strongly associated and also emerged as two partly overlapping concepts. We conclude that mental health educational institutions and supervisors could pay extra attention to the aspects of gender and work situation concerning mental health nurses' mentalizing and empathy in patient relations.

## 1 Introduction

In mental healthcare, clinicians' empathy as well as mentalizing abilities are associated with better opportunities to establish positive therapeutic relationships with patients (Priebe et al. 2019; Fonagy and Allison 2014). Both concepts are considered as partly overlapping domains by Choi‐Kain and Gunderson (2008), as they both involve the acknowledgement of mental states in other persons. According to these authors, empathy is more other‐orientated, while mentalizing is equally self‐ and other‐orientated.

Empathy is defined as the capacity to be affected by and share the emotional state of another, assess the reasons for the other's state, and identify with the other, adopting his or her perspective. It allows people to quickly and automatically relate to the emotional state of others (De Waal 2008). Empathy develops throughout early childhood and is shaped by a range of factors, including genetics, temperament, context, and environment (Decety and Holvoet 2021). Psychiatric disorders affiliated with a deficiency in empathy include psychopathy, antisocial, borderline, and narcissistic personality disorders; autism spectrum disorders; and schizophrenia (Decety and Moriguchi 2007; Rum and Perry 2020).

Mentalizing is described as the mental process by which individuals explain and predict behaviour by attributing mental states to themselves and others, such as thoughts, desires, feelings, and intentions (Fonagy and Target 2006; Decety 2020). The capacity to mentalize is obtained progressively over the course of the first years of life in the context of secure child‐carer relationships (Bateman and Fonagy 2013). It evolves in the context of interactions with others and is assumed to be continually influenced by the capacity to mentalize of those others (Luyten et al. 2020). Mentalizing helps to facilitate the regulation of affect and distress (Fonagy et al. 2017), particularly distress caused by interpersonal problems (Schwarzer et al. 2023). Excessive arousal and stress can lead to temporary lapses in mentalizing in non‐clinical as well as in clinical populations. Chronic impairments in mentalizing are implied in a wide range of psychiatric disorders, such as (borderline) personality disorder, depressive and anxiety disorders, eating disorders, somatoform disorders, autism spectrum disorder, substance abuse disorder, pathological gambling, attention‐deficit/hyperactivity disorder, psychotic disorders, and posttraumatic stress disorder (Luyten et al. 2020).

In the general population, factors associated with higher levels of empathy are female gender and having more education (Sommerlad et al. 2021). Clinicians' empathy has been studied mainly among professionals in general healthcare and to a lesser degree among mental healthcare workers, including mental health nurses. Higher levels of empathy among healthcare and/or social workers have been found to be associated with higher educational levels (Kuo et al. 2012), female gender, older age, and more years of work experience (Bogiatzaki et al. 2019; Moudatsou et al. 2020; Stanley, Mettilda Buvaneswari, and Meenakshi 2020). According to Salazar et al. (2023), healthcare workers' empathy towards patients decreases when confronted with patients' violent behaviour. This is supported by a study of Lindgren, Molin, and Graneheim (2021), who found that nursing staff of psychiatric inpatientwards experienced difficulties feeling empathy for patients with self‐harming behaviour. Additionally, mental health nurses who are less able to regulate their emotions when empathically engaging with patients may be at risk for compassion fatigue and burnout (Hunt, Denieffe, and Gooney 2017).

Healthcare professionals' mentalizing has been the subject of research to a smaller extent than empathy. It has been studied primarily in the field of psychology. Research on mental health nurses' mentalizing is also scarce. Factors associated with higher levels of mentalizing in the general population are female gender, younger age, and higher educational level (Vera Cruz et al. 2024; Pardini and Nichelli 2009; Li et al. 2013), and, among psychotherapists, female gender, working in private practice in comparison to a psychiatric inpatient clinic, and more years of work experience (Goldfeld et al. 2008; Steinmair, Richter, and Löffler‐Stastka 2020; Maheux et al. 2016). In the study of Steinmair, Richter, and Löffler‐Stastka (2020), it is suggested that the care setting and organisational variables of the psychiatric inpatient clinic (i.e., social context, working conditions, working hours, and patient selection) might have influenced the further development of the participants' mentalizing skills. According to the authors, these results also suggest that psychotherapists with higher mentalizing capacities as related to self‐consciousness might prefer working in private practice.

The disruption of healthcare professionals' mentalizing is a common feature of working with patients with challenging behaviours and distinctly impaired mentalizing capacities (Fonagy et al. 2017).

Mental health nurses constitute the largest workforce in mental healthcare (Fricchione et al. 2012; Organisation for Economic Co‐operation and Development (OECD) 2013). They work most closely with patients on a day‐to‐day basis and have a central position in the delivery of mental health services. Mental health nurses are on the front line when facing the frequently challenging behaviours of people with severe mental illness. This may be especially the case for mental health nurses working in acute mental health settings (Jenkins and Elliott 2004; Currid 2008).

According to Peplau (1992), the central feature of nursing practice in mental healthcare is the nurse–patient relationship. Through this relationship, processes of behaviour change that maximise patients' health are initiated. Within this relationship, nurses' communication skills, such as mentalising and empathising, might enable nurses to truly understand patients' needs. Furthermore, understanding a patient's situation from his/her perspective (i.e., mentalising) and experiencing congruent emotions to their situation (i.e., empathising) are considered important determinants of patients' recovery (Gerace 2020). Since the direct daily involvement of mental health nurses with patients' challenging behaviour, it might be extra important for these professionals to recognise their own difficulties with empathy and mentalizing, and be sensitive to the communicative implications this may have. It might also be important for mental health nurses to perceive patients' disrupted empathy and mentalizing, in order to build and sustain therapeutic relationships with these patients. A positive finding from research is that both empathy and mentalizing can be trained in healthcare workers (Paulus and Meinken 2022). In a systematic review of Free et al. (2024), intervention strategies designed to improve the mentalizing capacities of (mental) healthcare professionals reported favourable results. Therefore, we expect that the results of our study could contribute to the frame of reference of methods in mental health nursing.

To our knowledge, little research has looked at empathy and mentalizing of mental health nurses. This study sought to address this gap in the literature. The purpose of this study was threefold. First, to gain more insight in the level of empathy and mentalizing capacities of mental health nurses working with patients with severe mental illness, because of the importance of these skills for establishing good working relationships with these patients. We specifically chose these nurses and this patient group because of the higher risk of nurses' decreased empathy and mentalising in the confrontation with the frequently challenging behaviours of people with severe mental illness. During the mental health nurses' selection process and training, attention is paid to aspects of empathy and mentalizing such as observing, gathering evidence on the way patients perceive the situation confronting them, visualising patients' mental state, and observing what is happening in the interpersonal nurse–patient relation (Peplau 1987; Gerace 2020). Second, in view of future training and supervision, we want to gain more insight into the association between these mental health nurses' level of empathy and mentalizing on the one hand and sociodemographic and work characteristics of these nurses on the other hand. Third, in the context of designing empathy and mentalizing training programs, we want to gain more insight into the extent to which these mental health nurses' empathy and mentalizing overlap with each other. To this end, we tested the following hypotheses. First, we expect that in our study, the level of empathy and mentalizing of mental health nurses will be high in comparison to reference groups consisting of adults studying on working in different contexts. Second, we expect that mental health nurses' higher level of empathy and mentalizing will be positively associated with female gender, years of work experience, age, and outpatient work setting. Third, we expect that the mental health nurses' levels of empathy and mentalizing will be significantly associated.

Therefore, the research questions for this study are as follows:

1. What is the level of empathy and mentalizing of mental health nurses working with patients with severe mental illness, compared with reference groups of adults studying or working in different contexts?
2. What is the association between sociodemographic and work characteristics (gender, age, work experience, educational level, work setting) of mental health nurses working with patients with severe mental illness on the one hand, and these nurses' level of empathy and mentalizing?
3. What is the association between the level of empathy and mentalizing of mental health nurses working with patients with severe mental illness?

## 2 Methods

### 2.1 Study Design and Sampling

This study used a cross‐sectional design in adherence with the Strengthening the Reporting of Observational Studies in Epidemiology (STROBE) statement (Von Elm et al. 2008).

Eligibility for participation in this study was based on working as a mental health nurse or social worker with patients with severe mental illness in either (*a*) a high‐intensive work setting in mental healthcare or (*b*) an intermittent‐intensive work setting in mental healthcare. In this study, for readability we chose to use the abbreviation ‘MHN/SW’ for the mental health nurses and social workers.

High‐intensive work settings included closed high and intensive care (HIC) wards and acute treatment units providing intensive home treatment (IHT). HIC aims to provide optimal treatment and safety while restoring and maintaining contact between staff, patients, and relatives and promoting crisis prevention (Voskes et al. 2021). IHT focuses on stabilising psychiatric crises in order to prevent hospitalisation or facilitate early hospital discharge (Barakat et al. 2021).

Intermittent‐intensive work settings comprised ambulatory treatment settings providing flexible assertive community treatment (FACT) or primary mental healthcare. FACT is a European adaptation of Assertive Community Treatment (ACT). FACT teams support individuals with severe mental illness in the community, implying flexible changes between intensive and less intensive provision of care (Brekke et al. 2023). Primary mental healthcare provides low‐intensity community mental healthcare for individuals with mild to severe mental illness together with primary care physicians (Beckers et al. 2022).

All work settings work with a mixed spectrum of diagnostic groups: patients with psychotic disorders, anxiety and/or mood disorders, and/or personality disorders.

The study was carried out in four HIC‐, two IHT‐ and fifteen FACT‐units of a regional institution for specialised mental healthcare and in six units of three mental health institutions providing primary mental healthcare, all situated in The Netherlands. All eligible professionals were invited to participate (*n* = 330). There were no exclusion criteria. All participants signed informed consent. Filling out the (e)Case Report Form took approximately 10 min. Recruitment of participants and data collection took place between June 2019 and September 2019.

### 2.2 Data Sources and Measures

#### 2.2.1 Demographic and Work Characteristics

MHN/SW demographic and work characteristics were assessed using a questionnaire within the (e)Case Report Form and included gender, age, work experience, educational level, and work setting. Age and years of work experience in mental health care were measured in years; educational level, was coded as 0 = vocational, 1 = bachelor/master; gender as 1 = male, 2 = female; and work setting as 1 = high‐intensive, 2 = intermittent‐intensive. The latter group was split into a category 1.1 (= primary intermittent‐intensive) and 1.2 (= specialist intermittent‐intensive) for an extra sensitivity analysis. This was done to check for a possible difference between primary mental healthcare and FACT regarding the mental health illness' level of severity of the individuals receiving care in these two work settings.

#### 2.2.2 Empathy

The 28‐item Empathy Quotient (EQ) (Groen et al. 2015) was used to investigate empathy. It is a self‐report measure based on the Empathy Quotient (EQ) of Baron‐Cohen and Wheelwright (2004). The EQ has three subscales: Cognitive Empathy (CE), Emotional Empathy (EE), and Social Skills (SS). The CE subscale measures the capacity to work out what another person is likely to be thinking. The EE subscale measures the ability to recognise and respond emotionally to the affective state of another person. The SS subscale measures the ability to maintain relationships and to understand what is socially appropriate behaviour in interactions with other people (Pepper et al. 2019). The EQ items are rated on a 4‐point Likert scale (strongly agree, slightly agree, slightly disagree, strongly disagree). According to Groen et al. (2015), the EQ has overall good validity. The internal consistency is also good with a Cronbach alpha coefficient of 0.89. The internal consistency of the subscales CE and EE is good with a Cronbach alpha coefficient of 0.89 and 0.80, respectively. The internal consistency of the SS subscale is poor with a Cronbach alpha coefficient of 0.57. The test–retest reliability of the EQ is good (*r*(58) = 0.74, *p* < 0.001). The test–retest reliability of the subscales varies from good for the subscales CE and SS (CE: *r*(58) = 0.74, *p* < 0.001; SS: *r*(58) = 0.76, *p* < 0.001) and somewhat less reliable for the subscale EE (*r*(58) = 0.58, *p* < 0.001). In the current study, the internal consistency of the 28‐item EQ was good with a Cronbach alpha coefficient of 0.83. The internal consistency of the subscales CE, EE, and SS varied from good to poor with Cronbach alphas of 0.87, 0.69, and 0.52, respectively.

#### 2.2.3 Mentalizing

The Mentalisation Scale (MentS) (Dimitrijević et al. 2017) was used to investigate mentalizing. The MentS is a 28‐item self‐report measure and is comprised of three subscales: MentS‐Other (MentS‐O) (10 items), MentS‐Self (MentS‐S) (8 items), and MentS‐Motivation (MentS‐M) (10 items). The MentS‐O subscale measures the capacity to understand others' behaviour in terms of intentional mental states. The MentS‐S subscale measures the capacity to understand one's own behaviour in terms of intentional mental states. The MentS‐M subscale measures one's need to understand the other person's and one's own behaviour in terms of intentional mental states. The MentS items are rated on a five‐point Likert scale, ranging from 1 (completely disagree) to 5 (completely agree). The MentS has good construct validity. The MentS' internal consistency is good with a Cronbach alpha coefficient of 0.84 (Dimitrijević et al. 2017). The internal consistencies for the three subscales are acceptable with a Cronbach alpha of 0.77 for MentS‐O and MentS‐M and 0.76 for MentS‐S. In the current study, internal consistency was examined at both the full scale and subscale levels. The Cronbach alpha coefficient of the MentS was 0.87. Cronbach alphas for the three subscales were 0.68 for MentS‐S, 0.78 for MentS‐O, and 0.80 for MentS‐M.

### 2.3 Data Analysis

Data were analysed with SPSS, version 28.0. Descriptive statistical analysis was used to assess the demographic characteristics (gender, age, work experience, educational level, and work setting) and the level of empathy and mentalizing of our sample of MHN/SW. Empathy and mentalizing were calculated separately for men and women. This was necessary to perform adequate comparisons with the external reference groups from the literature since the reference populations' outcomes were also divided into male and female subgroups. From empirical research, one reference group was available for empathy (Groen et al. 2015) and also one for mentalizing (Dimitrijević et al. 2017). Then, independent t‐tests with a 95% CI were run for empathy on the EQ (and its subscales) for our sample and a first reference group from research by Groen et al. (2015). This reference group consisted of 685 adults of a community sample recruited through the social networks of the researchers of Groen et al. (2015) and psychology students collaborating on their research project. Next, independent t‐tests with a 95% CI were run to test for significant differences on the MentS (and its subscales) between our sample and a second external reference group from research by Dimitrijević et al. (2017). In this case, the reference group consisted of 566 adult employees at various positions within a dairy production factory and adult college freshmen engaged in various fields of study (Dimitrijević et al. 2017). Effect sizes for the difference between our sample and the reference groups were calculated using Cohen's *d*.

To measure the association between MHN/SW levels of empathy and mentalizing on the one hand, and MHN/SW demographic characteristics on the other, after checking for interaction effects between these variables, standard multiple regression analyses using the enter method were executed.

Finally, the relationship between empathy and mentalizing of the study sample of MHN/SW was investigated using the Pearson product–moment correlation coefficient. To detect the maximum shared variance between the MHN/SW level of empathy and mentalizing, the coefficient of determination (*R*) was calculated as the square of the highest correlation coefficient (*r* ^2^). Statistical significance was set at 5%.

### 2.4 Ethical Considerations

This study belongs to a broader research project considering the subject of mentalization. The study was approved by the Scientific Quality Committee of the Amsterdam Public Health Research Institute (Protocol Number: SQC2019‐061, date: 18th September 2019).

## 3 Results

### 3.1 Mental Health Nurses' Demographic and Work Characteristics

One hundred and eight MHN/SW gave written informed consent. One participant had to be excluded from the analysis because no gender was specified in the (e)Case Report Form. So, 107 MHN/SW participated in the study, resulting in a response rate of 32.4% within the group of (*n* = 330) invited professionals. The demographic characteristics of the response sample are described in Table 1.

**TABLE 1:** Mental health nurses' demographic and work characteristics (*n* = 107).

	Mental health nurses (*n* = 107)
	%/Mean (SD)
Gender^a^	
Female	65.7%
Male	33.3%
Age	45.3 (12.8)
Educational level	
Vocational	20.4%
Bachelors’/master	79.6%
Work setting	
High‐intensity	35.2%
Intermittent‐intensive	64.8%
Work experience (years)	19.4 (12.6)
Professional background	
Mental health nurse	81.5%
Mental health social worker	13.0%
Other	5.6%
Working hours (weekly)	30.7 (5.2)

Abbreviation: SD, standard deviation.

^a^ One participant gender not specified.

### 3.2 Level of Empathy of the Mental Health Nurses and Comparisons With a Reference Group of a Community Sample of Groen et al. (2015)

Both male and female MHN/SW had statistically significantly higher total scores on the EQ than the male (*p* = 0.042) and female (*p* = 0.026) participants of the reference group consisting of a community sample of Groen et al. (2015) (Table 2). The effect size, measured by Cohen's *d*, was *d* = 0.4 for the males and *d* = 0.3 for the females, both indicating small effects. Specifically, the male nurses had significantly higher scores on the subscale Emotional Empathy than the male participants of the reference group (*p* = 0.036). The effect size was *d* = 0.4, indicating a small effect. On the subscale Social Skills, the female nurses had significantly higher scores than the female participants of the reference group (*p* = 0.003). The effect size was 0.4, indicating a small effect.

**TABLE 2:** Means (SD) of the EQ total score and its subscales and group comparisons between mental health nurses (MHN) and the reference group of the community sample of Groen et al. (2015) (REF).

	Males				Females			
	MHN	REF	Group comparison		MHN	REF	Group comparison	
	*n* = 36	*n* = 270			*n* = 70	*n* = 415		
	Mean (SD)	Mean (SD)	Cohen's *d* (95% CI)	*p*	Mean (SD)	Mean (SD)	Cohen's *d* (95% CI)	*p*
Cognitive empathy	13.3 (4.8)	11.9 (4.8)	0.3 (−0.1, 0.6)	0.101	15.1 (3.9)	14.1 (4.7)	0.2 (−0.0, 0.5)	0.093
Emotional empathy	11.6 (3.3)	10.0 (4.4)	0.4 (0.0, 0.7)	0.036	14.7 (3.1)	14.3 (3.8)	0.1 (−0.1, 0.4)	0.404
Social skills	7.3 (2.2)	6.9 (2.6)	0.2 (−0.2, 0.5)	0.379	8.4 (2.0)	7.5 (2.4)	0.4 (0.1, 0.6)	0.003
EQ total score	32.3 (8.5)	28.8 (9.8)	0.4 (0.0, 0.7)	0.042	38.3 (6.4)	35.9 (8.6)	0.3 (0.0, 0.5)	0.026

Abbreviations: CI, confidence interval; *p*, *p*‐value; SD, standard deviation.

### 3.3 Level of Mentalizing of the Mental Health Nurses and Comparisons With a Reference Group of a Community Sample of Dimitrijević et al. (2017)

Both male and female MHN/SW had statistically significantly higher total scores on the MentS than the male (*p* = 0.001) and female (*p* < 0.001) participants of the reference group consisting of a community sample of Dimitrijević et al. (2017) (Table 3). The effect size, measured by Cohen's *d*, was *d* = 0.6 for the males and *d* = 0.7 for the females, both indicating medium effects. On the subscale MentS‐Motivation, the male and female MHN/SW also had significantly higher scores than the male (*p* < 0.001) and female (*p* < 0.001) participants of the reference group. The effect size, measured by Cohen's *d*, was *d* = 0.9 for the males, indicating a large effect, and *d* = 0.6 for the females, indicating a medium effect. Furthermore, the female MHN/SW had significantly higher scores on the subscale MentS‐Self than the female participants of the reference group (*p* < 0.001). The effect size, measured by Cohen's *d*, was *d* = 0.9, indicating a large effect.

**TABLE 3:** Means (SD) of the MentS total score and its subscales and group comparisons between mental health nurses (MHN) and the reference group of the community sample of Dimitrijević et al. (2017) (REF).

	Males				Females			
	MHN	REF	Group comparison		MHN	REF	Group comparison	
	*n* = 36	*n* = 237			*n* = 70	*n* = 329		
	Mean (SD)	Mean (SD)	Cohen's *d* (95% CI)	*p*	Mean (SD)	Mean (SD)	Cohen's *d* (95% CI)	*p*
MentS self	30.1 (4.1)	28.5 (5.7)	0.3 (−0.1, 0.6)	0.106	31.7 (3.7)	27.1 (5.4)	0.9 (0.6, 1.2)	< 0.001
MentS other	38.4 (3.7)	37.4 (4.9)	0.2 (−0.1, 0.6)	0.241	39.8 (4.0)	39.4 (4.5)	0.1 (−0.2, 0.3)	0.492
MentS motivation	40.5 (4.4)	35.6 (5.4)	0.9 (0.6, 1.3)	< 0.001	42.5 (4.3)	39.5 (4.9)	0.6 (0.4, 0.9)	< 0.001
MentS total score	109.1 (10.6)	101.5 (12.3)	0.6 (0.3, 1.0)	0.001	114.1 (9.3)	106.2 (11.3)	0.7 (0.5, 1.0)	< 0.001

Abbreviations: CI, confidence interval; *p*, *p*‐value; SD, standard deviation.

### 3.4 Associations Between Demographic Characteristics and Empathy of Mental Health Nurses

Multivariable linear regression was used to identify determinants of MHN/SW empathy. The analysis showed that female gender was the only variable from our variable‐set that statistically significantly related to the EQ total score (*β* = 0.36, *p* < 0.001), and also to the EQ subscales Cognitive Empathy (*β* = 0.20, *p* = 0.03), Emotional Empathy (*β* = 0.41, *p* < 0.001), and Social Skills (*β* = 0.21, *p* = 0.02) (Table 4). No significant associations were found for MHN/SW empathy with age, years of work experience, educational level, and work setting.

**TABLE 4:** Parameter estimates of multivariable linear regression models regressing the EQ total score (and its subscales) on demographic characteristics of mental health nurses' (*n* = 106).

Variables	*B*	SE	*β*	*t*	*p*
EQ total score					
Constant	30.68	5.05		6.06	< 0.001
Male versus female gender	5.88	1.51	0.36	3.89	< 0.001
Age (years)	−0.10	0.10	−0.18	−1.03	0.30
Work‐experience (years)	0.07	0.11	0.12	0.71	0.47
Vocational versus bachelor/master	−1.32	1.82	−0.07	−0.72	0.46
High‐intensive versus intermittent‐intensive work setting	0.19	1.62	0.01	0.11	0.90
Model summary: *R* ^2^ = 0.147, *R* ^2^ adj = 0.104					
Cognitive empathy					
Constant	15.67	2.92		5.35	< 0.001
Male versus female gender	1.83	0.87	0.20	2.09	0.03
Age (years)	−0.04	0.06	−0.13	−0.76	0.44
Work‐experience (years)	0.02	0.06	0.06	0.37	0.71
Vocational versus bachelor/master	−1.79	1.05	−0.16	−1.70	0.09
High‐intensive versus intermittent‐intensive work setting	−0.69	0.93	−0.07	−0.73	0.46
Model summary: *R* ^2^ = 0.081, *R* ^2^ adj = 0.035					
Emotional empathy					
Constant	7.67	2.21		3.46	< 0.001
Male versus female gender	3.07	0.66	0.41	4.64	< 0.001
Age (years)	−0.02	0.04	−0.07	−0.46	0.64
Work‐experience (years)	0.02	0.04	0.07	0.40	0.68
Vocational versus bachelor/master	1.14	0.79	0.13	1.43	0.15
High‐intensive versus intermittent intensive work setting	0.37	0.71	0.05	0.53	0.59
Model summary: *R* ^2^ = 0.204, *R* ^2^ adj = 0.164					
Social skills					
Constant	7.36	1.45		5.06	< 0.001
Male versus female gender	0.97	0.43	0.21	2.23	0.02
Age (years)	−0.04	0.03	−0.25	−1.38	0.17
Work‐experience (years)	0.03	0.03	0.21	1.13	0.25
Vocational versus bachelor/master	−0.66	0.52	−0.12	−1.27	0.20
High‐intensive versus intermittent‐intensive work setting	0.49	0.46	0.11	1.06	0.28
Model summary: *R* ^2^ = 0.093, *R* ^2^ adj = 0.048					

Abbreviations: *B*, unstandardized coefficients; *p*, *p*‐value; SE, standard error; *t*, *t*‐test statistic; *β*, standardised coefficients.

### 3.5 Associations Between Demographic Characteristics and Mentalizing of Mental Health Nurses

Multivariable linear regression was used to identify determinants of MHN/SW mentalizing. The analysis showed that from our variable‐set, female gender was statistically significantly related to the MentS total score (*β* = 0.22, *p* = 0.02) and also to the subscales MentS‐Self (*β* = 0.19, *p* = 0.05) and MentS‐Motivation (*β* = 0.18, *p* = 0.05) (Table 5). The analysis also showed that intermittent‐intensive work setting (primary mental healthcare and FACT) were statistically significantly related to the subscale MentS‐Motivation (*β* = 0.24, *p* = 0.01). The extra sensitivity analysis showed that this higher score on the subscale MentS‐Motivation was statistically significantly related to the FACT work setting (*β* = 0.26, *p* < 0.05) and not to the primary mental healthcare work setting. No statistically significant associations were found for age, years of work experience, and educational level.

**TABLE 5:** Parameter estimates of multivariable linear regression models regressing the MentS total score (and its subscales) on demographic characteristics of mental health nurses (*n* = 106).

Variables	*B*	SE	*β*	*t*	*p*
MentS total score					
Constant	105.45	6.79		15.52	< 0.001
Male versus female gender	4.66	2.03	0.22	2.29	0.02
Age (years)	−0.04	0.14	−0.06	−0.33	0.73
Work‐experience (years)	−0.00	0.14	−0.00	−0.01	0.99
Vocational versus bachelor/master	−3.73	2.44	−0.15	−1.52	0.13
High‐intensive versus intermittent‐intensive work‐setting	2.63	2.18	0.12	1.21	0.22
Model summary: *R* ^2^ = 0.086, *R* ^2^ adj = 0.040					
MentS‐other					
Constant	39.45	2.72		14.46	< 0.001
Male versus female gender	1.31	0.81	0.15	1.60	0.11
Age (years)	−0.04	0.05	−0.13	−0.72	0.46
Work‐experience (years)	0.01	0.05	0.04	0.23	0.81
Vocational versus bachelor/master	−1.46	0.98	−0.15	−1.49	0.13
High‐intensive versus intermittent‐intensive work‐setting	0.28	0.87	0.03	0.32	0.75
Model summary: *R* ^2^ = 0.053, *R* ^2^ adj = 0.005					
MentS‐self					
Constant	29.47	2.66		11.04	< 0.001
Male versus female gender	1.58	0.79	0.19	1.98	0.05
Age (years)	−0.01	0.05	−0.06	−0.34	0.72
Work‐experience (years)	0.06	0.05	0.19	1.06	0.29
Vocational versus bachelor/master	−1.65	0.96	−0.17	−1.72	0.08
High‐intensive versus intermittent‐intensive work‐setting	0.06	0.85	0.00	0.08	0.93
Model summary: *R* ^2^ = 0.093, *R* ^2^ adj = 0.047					
MentS‐motivation					
Constant	36.50	2.98		12.23	< 0.001
Male versus female gender	1.77	0.89	0.18	1.98	0.05
Age (years)	0.01	0.06	0.03	0.21	0.83
Work‐experience (years)	−0.07	0.06	−0.21	−1.19	0.23
Vocational versus bachelor/master	−0.59	1.07	−0.05	−0.55	0.57
High‐intensive versus intermittent‐intensive work‐setting	2.29	0.95	0.24	2.39	0.01
Model summary: *R* ^2^ = 0.106, *R* ^2^ adj = 0.061					

Abbreviations: *B*, unstandardized coefficients; *p*, *p*‐value; SE, standard error; *t*, *t*‐test statistic; *β*, standardised coefficients.

### 3.6 The Association Between Empathy and Mentalizing of Mental Health Nurses

Within our sample of MHN/SW, there were moderate to strong, positive and significant correlations between the scores on the EQ and its subscales Cognitive Empathy and Emotional Empathy on the one hand, and the MentS and its three subscales MentS‐Self, MentS‐Other and MentS‐Motivation on the other hand (correlation coefficients varying from 0.35 to 0.66) (Table 6). Correlations between the MentS and the subscale Social Skills of the EQ were also positive and significant but remarkably smaller, with a correlation coefficient of 0.26. Empathy and mentalizing emerged as partly overlapping concepts within this sample with a shared variance between level of empathy and mentalizing of 43% (*r* ^2^ = 0.43). As a post hoc analysis, we checked whether specific dimensions of mentalizing (with the use of the MentS subscales) would be associated with empathy (according to the EQ) within our sample of MHN/SW. We assumed that the EQ would be mainly associated with the subscale MentS‐Other. Correlations for the EQ and the subscales MentS‐Self, MentS Other and MentS‐Motivation were all significant and varied between 0.46, 0.59, and 0.55, respectively. The proportion of shared variance of the EQ and the MentS subscales was highest for MentS Other (0.34) and MentS‐Motivation (0.30) and somewhat lower for MentS‐Self (0.21). The correlation between the observed total scales of the EQ and MentS was high (0.66).

**TABLE 6:** Pearson correlations between mental health nurses' (*n* = 107) mpathy (according to the EQ and its subscales) and mentalizing (according to the mentS and its subscales).

	Cognitive empathy	Emotional empathy	Social skills	EQ
MentS‐self	0.38**	0.35**	0.28**	0.46**
MentS‐other	0.58**	0.46**	0.19*	0.59**
MentS‐motivation	0.45**	0.56**	0.17	0.55**
MentS	0.58**	0.57**	0.26**	0.66**

* *p* < 0.05.

** *p* < 0.01 (two‐tailed).

## 4 Discussion

This study focused on MHN/SW empathy and mentalizing. As expected, our main results demonstrated that male and female MHN/SW had higher levels of empathy and mentalizing than male and female participants of two external reference groups. After checking for interaction effects between variables, we also found, corresponding to other studies, that female gender was positively associated with higher levels of empathy (Bogiatzaki et al. 2019; Stanley, Mettilda Buvaneswari, and Meenakshi 2020) and mentalizing (Goldfeld et al. 2008). Furthermore, we found, comparable to the study of Steinmair, Richter, and Löffler‐Stastka (2020), that an intermittent‐intensive/ambulatory (not high‐intensive/acute) work setting was positively associated with a higher score on the subscale MentS‐Motivation. An extra sensitivity analysis showed that this higher score was specifically significantly related to the FACT work setting and not to the primary mental healthcare work setting. Moreover, empathy and mentalizing of MHN/SW were moderately to strongly associated in our study.

It is possible that the MHN/SW higher levels of empathy and mentalizing is associated with job (self‐) selection processes. Individuals with higher levels of empathy and mentalizing might have a preference for working as a mental health nurse or receive encouragement from others to do so. Notwithstanding the MHN/SW higher levels of empathy and mentalizing, despite training, supervision, and on‐the‐job training, it is possible that empathy and mentalizing skills are disrupted in challenging interactions with patients. Also, more work experience in mental healthcare may increase MHN/SW empathy and mentalizing capacities, even though this was not significant in our study.

Within our sample of MHN/SW, female gender was related to higher levels of empathy and mentalizing. This corresponds with previous studies indicating that women generally have higher levels of empathy compared to men (Andersen et al. 2020). However, it is possible that gender differences in self‐reported empathy, such as the EQ, are induced by a female tendency to report stronger empathic responses, rather than actual differences in male and female empathic capacity (Löffler and Greitemeyer 2021). Intermittent‐intensive work setting were related to a higher score on the subscale MentS‐Motivation in our study. This subscale measures one's need to understand the other person's and one's own behaviour in terms of intentional mental states. It is possible that MHN/SW working in intermittent‐intensive/ambulatory work settings experience less excessive stress in their interactions with patients, compared with MHN/SW working in high‐intensive/acute work settings. This may result in an increase in the motivation to mentalize for nurses working in intermittent‐intensive work settings. In contrast, MHN/SW working in high‐intensity work/ acute settings more often deal with patients' aggressive behaviour on a daily basis. Possibly these professionals use a more directive than mentalizing approach in order to regain safety and avoid damage to patients, themselves, and the material environment. Furthermore, in a study of Hsiao, Lu, and Tsai (2015), mental health nurses of acute wards showed more negative attitudes towards individuals with mental illness compared with those working in psychiatric rehabilitation units, outpatient clinics, and community psychiatric rehabilitation centers. Also, in a study of Bodner et al. (2015), mental health nurses' negative attitudes towards individuals with borderline personality disorder were associated with decreased empathy of these nurses.

We further found that age was not associated with a higher level of empathy in our study. According to Beadle and De La Vega (2019), findings in the literature on the relationship between age and empathy are mixed, possibly due to the large variation in the behavioural and neuroimaging methods used to study empathy. It is plausible that our sample of MHN/SW may have started with already higher‐than‐average levels of empathy due to the professional selection processes, partly causing a ceiling effect.

Empathy and mentalizing of MHN/SW were moderately to strongly associated in our study. Correlations in the post hoc analysis for the EQ and the subscales MentS‐Self, MentS‐Other, and MentS‐Motivation were all significant, which is largely in line with our assumptions. The proportion of shared variance of the EQ and the MentS subscales was highest for MentS‐Other and MentS Motivation, and somewhat lower for MentS‐Self. This also fits in with our assumption. Notice that the correlation between the observed total scales of the EQ and MentS was high, suggesting a large overlap. The idea of empathy and mentalizing as two partly overlapping concepts, as suggested by Choi‐Kain and Gunderson (2008), can be largely confirmed. In contrast to this, in a study of Kanske et al. (2016), empathy and mentalizing of non‐clinical individuals during social interactions were independent, both on a behavioural and neural level. In this study, empathy also inhibited mentalizing‐related activity in highly emotional situations, thus harming mentalizing performance. According to these authors, highly emotional situations can lead to impaired mentalizing in real‐life social interactions. They also recommend that training programs aimed at improving social skills such as empathy and mentalizing should consider that improving empathy may not necessarily enhance mentalizing capacities, and vice versa.

### 4.1 Strengths and Limitations

Our study has several strengths. First of all, this study was the first to examine the empathy and mentalizing abilities of mental health nurses, the association between mental health nurses' level of empathy and mentalizing on the one hand and sociodemographic and work characteristics of these nurses on the other, and the association between mental health nurses' empathy and mentalizing. Second, we did a sufficiently powered study with a sample of 107 mental health nurses from different work settings in which empathy and mentalizing can come under pressure, with a decrease of these crucial skills as a possible result. Third, we used validated measures to assess empathy and mentalizing. Fourth, we compared the mental health nurses' level of empathy and mentalizing to reference groups from very different contexts. This allowed us a crude test of our hypotheses.

This study has some limitations. First, we used self‐report measures, which can result in response bias, involving an overestimation of nurses' own skills. Also, research shows that self‐report and behavioural measurements of empathy are often weakly associated. This can be explained by a discrepancy between belief‐based self‐perceptions and situation‐specific behaviours concerning one's empathy (Sunahara et al. 2022). Second, due to the heterogeneity of the external reference groups, the interpretation of our findings regarding the comparison between the MHN/SW level of empathy and mentalizing and these reference groups can be biased. Third, the mental health nurses' levels of empathy and mentalizing were compared with those of reference groups from very different contexts. Context is part of the working environment that shapes attitudes and learning of people as they acquire the skills necessary to study and work. In our study, context may influence reported levels of empathy and mentalization. However, when directly comparing different groups, such as we did, it is not possible to control for contextual differences. Fourth, the internal consistency of the EQ‐subscale Social Skills was poor. According to Groen et al. (2015), this could be due to the lower number of items (6) of this subscale compared to the EQ subscales Cognitive Empathy (11 items) and Emotional Empathy (11 items). According to these authors, the poor internal consistency of the EQ subscale social skills could also be due to the fact that it largely consists of reversed items, which have been shown to have overall lower consistency than forward items. The relatively low consistency of the Social Skills subscale would render it less suitable to study for instances of correlations of the subscale with other variables within the empathy domain or with other variables. Lack of consistency usually means that the scale includes relatively much ‘noise,’ which does not correlate with anything and reduces the ability of the scale to detect correlations that are present. Fifth, the response rate of 32.4% for the MHN/SW was quite low. It is possible that the MHN/SW with higher levels of empathy and mentalizing abilities may be more likely to participate in the study, given their affinity for the subject of study. This could affect the generalisability of the results. However, this same bias may also apply to the control samples, limiting the effects on comparisons.

## 5 Conclusions

This study demonstrated that mental health nurses had higher levels of empathy and mentalizing than reference groups consisting of community samples. Mental health nurses' demographic characteristics positively associated with their level of empathy and mentalizing were female gender (for empathy and mentalizing) and an intermittent‐intensive/ambulatory work setting in contrast to a high‐intensive/acute setting (for the subscale ‘Motivation’ of the measure used in this study to assess mentalizing). Empathy and mentalizing of mental health nurses were strongly associated in our study and also emerged as two partly overlapping concepts. For future research, it is important to assess mental health nurses' empathy and mentalizing during challenging interactions with patients. For this purpose, it is important to develop measurement tools to assess health care professionals' empathy and mentalizing during such difficult nurse–patient interactions. Furthermore, for future research, studies with an experimental design are needed to examine the implementation and effectiveness of training courses aimed at promoting mental health nurses' empathy and mentalizing, especially in challenging interactions with patients.

## 6 Relevance to Clinical Practice

In real‐life social interactions, such as the interactions between mental health nurses and individuals with severe mental illness, highly emotional situations can lead to a decrease in empathy and mentalizing. Nursing education programs should aim to promote mental health nurses' empathy and mentalizing, specifically in challenging interactions with patients. In relation to empathy and mentalizing in challenging nurse–patient interactions, training courses for mental health nurses could pay extra attention to the aspects of gender and work situation, specifically the acute mental health setting. Training courses could be aimed at promoting empathy and mentalizing of nurses working in acute inpatient settings.

## Author Contributions

All authors had full access to the data in the study and take responsibility for the integrity of the study procedure. All authors reviewed the results and approved the final version of the manuscript. The authors confirm the following specific contribution to the paper: study conception and design: G.F., W.S., and B.v.M.; data collection: G.F. and W.S.; analysis and interpretation of results: G.F., W.S., B.v.M., and A.H.; draft manuscript preparation: G.F., W.S., B.v.M., A.B., and A.H.

## Ethics Statement

This study belongs to a broader research project considering the subject of mentalisation and the study was approved by the Scientific Quality Committee of the Amsterdam Public Health research institute (Protocol Number: SQC2019‐061, date: 18th September 2019).

## Conflicts of Interest

The authors declare no conflicts of interest.

## Data Availability

The data that support the findings of this study are available on request from the corresponding author. The data are not publicly available due to privacy or ethical restrictions.
